# Study of the Chemical Vapor Deposition of Nano-Sized Carbon Phases on {001} Silicon

**DOI:** 10.3390/ma16227190

**Published:** 2023-11-16

**Authors:** Teodor Milenov, Dimitar Trifonov, Dobromir A. Kalchevski, Stefan Kolev, Ivalina Avramova, Stoyan Russev, Kaloyan Genkov, Georgi Avdeev, Dimitar Dimov, Desislava M. Karaivanova, Evgenia Valcheva

**Affiliations:** 1 “Academician E. Djakov” Institute of Electronics, Bulgarian Academy of Sciences, 72 Tzarigradsko Chaussee Blvd., 1784 Sofia, Bulgaria; dtriffonoff@gmail.com (D.T.); dobromirak@gmail.com (D.A.K.); skkolev@ie.bas.bg (S.K.); dadimov@ie.bas.bg (D.D.); dkaraivanova@ie.bas.bg (D.M.K.); 2 Institute of General and Inorganic Chemistry, Bulgarian Academy of Sciences, Acad. G. Bonchev Str., bl. 11, 1113 Sofia, Bulgaria; iva@svr.igic.bas.bg; 3 Faculty of Physics, Sofia University “St. Kliment Ohridski”, 5 James Bourchier Blvd., 1164 Sofia, Bulgaria; scr@phys.uni-sofia.bg (S.R.); kgenkov@uni-sofia.bg (K.G.); epv@phys.uni-sofia.bg (E.V.); 4 “Academician Rostislav Kaishev” Institute of Physical Chemistry, Bulgarian Academy of Sciences, Acad. G. Bonchev Street, bl. 11, 1113 Sofia, Bulgaria; g_avdeev@ipc.bas.bg

**Keywords:** chemical vapor deposition, carbon phases, diamond, silicon carbide, nanomaterials, thin films

## Abstract

Different nano-sized phases were synthesized using chemical vapor deposition (CVD) processes. The deposition took place on {001} Si substrates at about 1150–1160 °C. The carbon source was thermally decomposed acetone (CH_3_)_2_CO in a main gas flow of argon. We performed experiments at two ((CH_3_)_2_CO + Ar)/Ar) ratios and observed that two visually distinct types of layers were deposited after a one-hour deposition process. The first layer type, which appears more inhomogeneous, has areas of SiO_2_ (about 5% of the surface area substrates) beside shiny bright and rough paths, and its Raman spectrum corresponds to diamond-like carbon, was deposited at a (CH_3_)_2_CO+Ar)/Ar = 1/5 ratio. The second layer type, deposited at (CH_3_)_2_CO + Ar)/Ar = a 1/0 ratio, appears homogeneous and is very dark brown or black in color and its Raman spectrum pointed to defect-rich multilayered graphene. The performed structural studies reveal the presence of diamond and diamond polytypes and seldom SiC nanocrystals, as well as some non-continuously mixed SiC and graphene-like films. The performed molecular dynamics simulations show that there is no possibility of deposition of sp^3^-hybridized on sp^2^-hybridized carbon, but there are completely realistic possibilities of deposition of sp^2^- on sp^2^- and sp^3^- on sp^3^-hybridized carbon under different scenarios.

## 1. Introduction

Carbon has many allotropic forms: 3D-ordered (diamond: 100% sp^3^-hybridized carbon and graphite: 100% sp^2^-hybridized carbon); 2D-ordered (graphene: 100% sp^2^-hybridized carbon); 1D-ordered (carbyne: 100% sp^1^-hybridized carbon); disordered (amorphous carbon (aC) and diamond-like carbon or tetrahedral carbon (taC). There are also different polymorphic phases of sp^2^-hybridized carbon fullerenes (the most popular for practical applications are C_60_ and C_70_), carbon nanotubes (single-walled carbon nanotubes (SWCNTs) as well as multiwalled carbon nanotubes (MWCNTs)), nanorods, etc. Chemical vapor deposition (CVD) is one of the preferred processes for the practical deposition of aC, taC, nano-/micro-diamond and graphene films (see [1,2,3,4]). The most widely used substrates for carbon film deposition are Si wafers (a widely available commercial product, which is isostructural to sp^3^-hybridized carbon).

On the other hand, the development of CVD methods for the deposition of carbon phases also led to the initial attempts to perform the so-called “silicon carbonization” of the Si substrate. These attempts were only partially successful. The layers were not continuous; there were many empty spaces under the layers and the adhesion was very poor. These results are fully explainable because Si and SiC have a serious difference in their lattice parameters, in the coefficients of their thermal expansion, etc. Later, Nishino et al. [5] proposed the multistep method from which many of the further CVD methods for the synthesis of SiC on Si originate: see Severino et al. [6,7]. In the mentioned methods, as well as in the epitaxial growth of Si, the surface of the Si substrate plays a crucial role as the presence of native SiO _2_ on the surface apparently does not allow the development of an epitaxial growth process on Si. Researchers have also noted the necessity of careful elimination of native SiO_2_, which apparently does not allow the development of an epitaxial growth process on Si. We should also mention that other developed methods for the synthesis of SiC on Si substrates using CVD rely on the decomposition of a CO precursor: see [8,9,10,11]. The authors of these methods assumed that most of the oxygen atoms leave the substrate/gas phase interface region as SiO while SiC, SiO_2_ and Si–O–C complexes remain in the layer, but further annealing at high temperature results in the evaporation of the oxygen-containing components into the gas phase.

In our earlier works, we have established that the deposition of thin carbon films using CVD via the decomposition of acetone ((CH_3_O)_2_CO) at temperatures above 1150 °C for 9–12 min leads to the formation of two different interlayers between the graphene film and the Si substrate: the first of which consists predominantly of a mixed phase of multilayered nanographene flakes, nanodiamond-/diamond-like carbon and C_70_ fullerenes; while the second one is dominated by nanographene flakes and C_70_ fullerenes, where the amount of C_70_ is greater than that of the former interlayer type (see [6,7,8,9,10,11,12,13]). In all cases, there was SiC in the samples, but its morphology was not established, nor was where it was located. The ongoing CVD process onto these interlayers resulted in the deposition of mixed carbon phases dominated either by sp^3^-hybridized carbon or by a graphene layer with some sp^3^-hybridized carbon content [12,13]. It has also been found (see ref. [14]) that under similar conditions, the growth of micro-sized diamond crystals is possible if there is a sufficient amount of stable nuclei of sp^3^-hybridized carbon. It should also be clearly noted that we have not found any other references for the parallel synthesis of SiC during the deposition of 2D and 3D carbon phases on Si substrates.

Our present work is focused on the study of the synthesis of nano-sized carbon phases at the silicon substrate thin film interface and the presence of an intermediate transition layer between the substrate and the deposited film. In addition, we aimed to trace and simulate the main interactions of the thermal dissociation products of acetone and phases of predominantly sp^2^- or sp^3^-hybridized carbon. In this sense, our main tasks were: (1) to clarify the most probable paths for the synthesis of ordered phases of sp^3^-hybridized carbon on films of ordered sp^2^-hybridized carbon; (2) to elucidate the process of deposition of carbon phases on Si substrate.

## 2. Materials and Methods

To solve the above tasks, we choose (i) to study the CVD processes of carbon phase deposition during the thermally stimulated decomposition of acetone in continuous processes with a longer duration (in the range of 60 to 90 min in different experimental setups) and two very different concentrations of the hydrocarbon precursor in the gas phase, and (ii) to simulate the deposition of sp^3^-hybridized carbon phases on graphene and defective graphene (a 2D sp^2^-hybridized carbon material), as well as on sp^3^-hybridized carbon.

**Figure 1:**
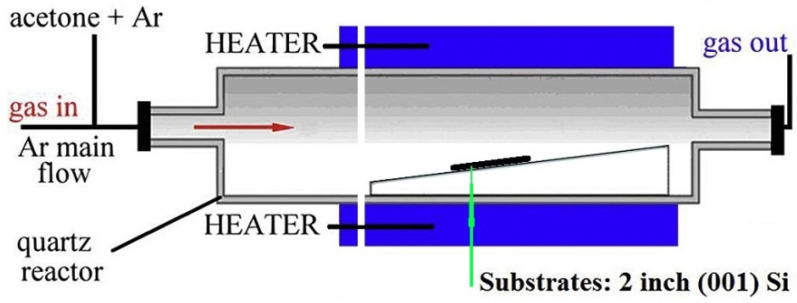
Schematic representation of the experimental setup for the CVD of carbon films on {001} Si substrates using the thermal decomposition of acetone.

The precursor (acetone) was chosen as it is not highly toxic, it has a relatively linear dependence of vapor wall pressure on temperature and it is high enough to provide a sufficient amount of incoming carbon to support the deposition process. In addition, it was expected that the presence of oxygen atoms in the acetone would help in the analysis of the deposition of the layer on the Si substrate: see the Introduction section). The gas flow ratio of Ar/(Ar + ((CH_3_)_2_CO) at 150/30 cm^3^/min is similar to that used for graphene deposition (see ref. [13]). The gas flow of acetone at 120 cm^3^/min without using a carrier gas (Ar) was used in order to obtain a significantly different result in comparison to the experiment where the precursor was ratioed with carrier gas. The experimental setup is shown schematically in Figure 1.

The gas system provides two gas flows mixed and fed into the reactor: the first is the carrier gas (Ar) with a flow rate of about 150 cm^3^/min; the second is a mixture (Ar + (CH_3_)_2_CO) from the thermostat with acetone at 15 °C with a flow rate of about 30 cm^3^/min. The deposition time was 45, 60 or 90 min, respectively. In order to distinguish the effect of a higher concentration of the gas phase with carbon on the carbon phases obtained, several experiments were performed using only one gas stream: that containing Ar + C_3_H_6_O at a flow rate of about 120 cm^3^/min (thermostat temperature of 18 °C) for a total deposition time of 60 min.

The structure of the obtained films was characterized using X-ray diffractometry and a Panalytical Empyrean apparatus (Malvern Panalytical, Malvern, UK). The measurements were performed with two geometry settings: powder X-ray diffractometry geometry (XRD) (θ-2θ) scans; and grazing incidence X-ray diffractometry (GIXRD) (ω-2θ) scans, respectively. The surface morphology of the specimens was examined using scanning electron microscopy (SEM) in both secondary electron and backscattered electron imaging (SE and BE, respectively) with a LYRA TESCAN (SEM) (TESCAN GROUP a.s., Brno, Czech Republic) or JEOL JSM-6390 microscope (JEOL Ltd., Tokyo, Japan) while the elemental analysis was performed using energy-dispersive X-ray analysis (EDX) and a Brucker QUANTAX 200 Spectrometer (Brucker Co., Elk Grove Village, IL, USA) or INCA X-Sight 7582 unit (Oxford Instruments, Abingdon, UK), respectively. The Raman measurements were carried out in backscattering geometry using a micro-Raman HORIBA Jobin Yvon (HORIBA Jobin Yvon, Longjumeau, France) LabRAM HR 800 visible spectrometer equipped with a Peltier-cooled CCD detector with He–Ne (633 nm wavelength and 0.5 mW) laser excitation. The 514 nm (about 23 mW), 488 nm (about 24 mW) as well as 456 nm (about 24 mW) lines of an external Ar laser were also used. The laser beam was focused on a spot of about 1 μm in diameter, the spectral resolution being about 1 cm^−1^ or better. The X-ray photoelectron spectra were obtained using unmonochromatized Al K_α_ (1486.6 eV) radiation in a VG ESCALAB (Thermo Fisher Scientific, Waltham, MA, USA) MK II electron spectrometer under a base pressure of 1 × 10^−8^ Pa. The spectrometer resolution was calculated from the Ag3d_5/2_ line with an analyzer transmission energy of 20 eV. The full width at half maximum (FWHM) of this line was 1 eV. The spectrometer was calibrated against the Au4f_7/2_ line (84.0 eV) and the samples’ charge was estimated from the C1s (285 eV) spectra from natural hydrocarbon contaminations on the surface. The accuracy of the binding energies (BEs) measured was 0.2 eV. The photoelectron spectra of the C1s, O1s and Si2p of the amorphous films deposited on the Si substrate were recorded and corrected by subtracting a Shirley-type background and quantified using the peak area and Scofield’s photoionization cross-sections.

All theoretical studies/calculations are performed using the software package CP2K/quickstep and have the GGA level of theory with the Perdew–Burke–Ernzerhof (PBE) functional. The double-zeta quality basis set Perdew–Burke–Ernzerhof (PBE), optimized for gas and condensed phase system properties, is applied to all atoms. The electronic wavefunction is modeled using the Gaussian Plane Wave (GPW) approach. Only the valence electrons are modeled explicitly. The core electronic shells are represented as Goedecker–Teter–Hutter (GTH) pseudopotentials, optimized for PBE. The charge density cutoff of the finest grid level is 400 Ry. There are five multigrids used. All calculations are performed using the unrestricted Kohn–Sham formalism. Dispersion interactions are accounted for in all calculations. The latest D3 revision of the dispersion-corrected density functional theory (DFT-D) method is used with a three-body term enabled. The reactions are modeled using Born–Oppenheimer metadynamics (MTD). The initial frame for each run is generated using either cell optimization or previous MTD calculations.

## 3. Results

### 3.1. Optical and SEM

**Figure 2:**
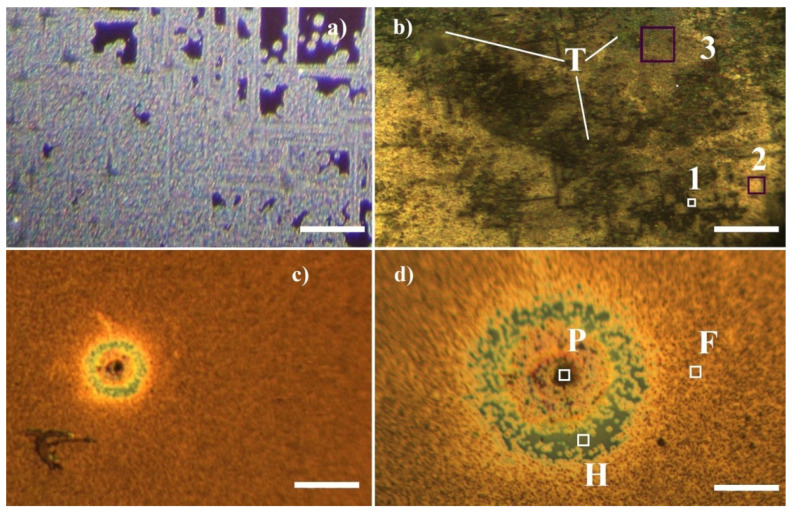
Optical microscopy images of (**a**) surface of as-deposited film I-type; (**b**) surface of I-type film, etched in HF solution in H_2_O; (**c**) a typical hillock-centered crater-type defect from II-type films; (**d**) magnified image from (**c**) panel. The points denoted by 1, 2 and 3 in the (**b**) panel indicate the positions at which the Raman spectra of I-type films were taken. The points denoted by H, P and F in the (**d**) panel indicate the positions at which the Raman spectra of II- type films were taken. The markers in (**a**–**c**) panels represent 300 µm, while the ones in the (**d**) panel represent 100 µm.

The CVD processes carried out with significantly different concentrations of the hydrocarbon precursor (in this case, acetone) lead to the formation of films with completely different morphologies:

- The first of these (Figure 2a) are films with two clearly distinguishable unevenly distributed areas: one is a flat and transparent film (most likely SiO_2_ with a thickness of about 100 nm according to the color of the transparent film: see, e.g., [15]) and occupies on average 5% of the surface area of the Si substrates; the other is bright, colorless and transparent, with an uneven relief surface, usually forming overlapping paths with an orientation along the <011> direction and a width of about 20–60 μm and roughly round islands of similar sizes, located in the areas covered with SiO_2_. The first of the described layers does not show a clear Raman spectrum, while the relief’s transparent areas have a clear Raman spectrum that varies in different positions (see Figure S1 (Supplementary Information File (SIF)) between that of the multilayer defective graphene and a spectrum of a more amorphized multilayer defective graphene with a fairly high content of sp^3^-hybridized carbon. These films will be denoted further as I-type.
- The second ones (Figure 2c,d) are brown- to black-colored smooth-looking films which possess a fine crystalline morphology with crystals of a sub-micron size, some of which appear black. Some typical hillock-centered crater-type defects characteristic of steady-state growth problems are observed (see Figure 2c,d). It should be clearly noted that a transparent film (most probably of SiO_2_) is always observed at the bottom of the craters while the remaining surface of the layers has a clear granular morphology, as the individual grains are up to several micrometers in size. These films will be denoted further as II-type.

Assuming that the blue/violet regions of the films deposited at a lower hydrocarbon precursor concentration (I-type, Figure 2a) contain some SiO_2_-based phases (as well as to ensure direct observation of the nanocrystalline phases formed on the substrate), these samples were etched for 30 s in a 15% solution of HF in bi-distilled water, then and washed in a 20% NaOH solution in bi-distilled water as well as in pure bi-distilled water. A larger part of the uneven, brightly shining, colorless and transparent relief part of the surface with clearly formed paths, 20–60 µm wide and with round islands with a similar diameter, does not change significantly under the influence of acid, and the relief is preserved. However, all areas colored in blue/violet, as well as some of the relief sections, are dissolved or detached, respectively, from the surface after this treatment. In the remaining sections, the morphology changes completely (see Figure 2b): the paths and islands have disintegrated into nanocrystalline phases and flakes. The surface there is visibly rough, with dark, almost black-colored areas visible in places, as well as transparent shiny areas (e.g., the areas marked T in Figure 2b). In addition, strip-shaped voids oriented parallel to <011> are observed on the surface black lines in Figure 2b.

**Figure 3:**
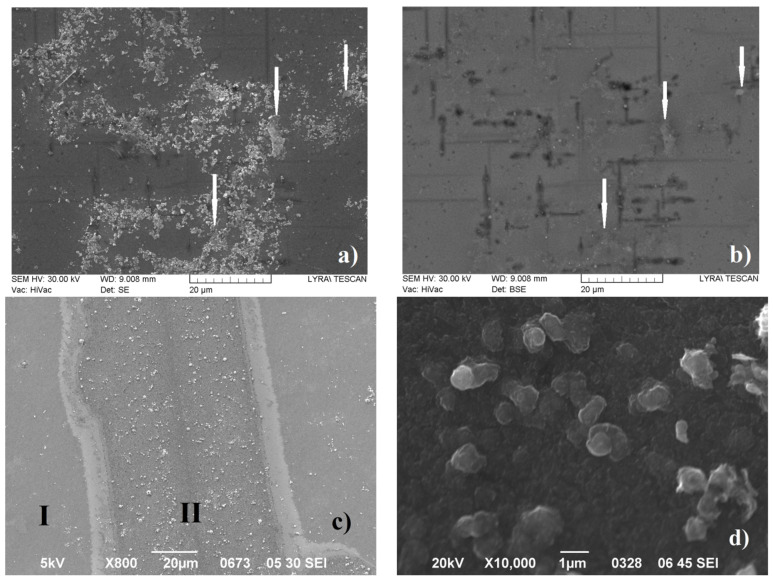
Overview of SEM images taken from HF-treated I-type films in SE (**a**) and BE (**b**) image modes, respectively, as well as a relief region that remains unresolved in SE image mode (**c**—panel). Surface morphology of a layer of type II taken in SEM SE image mode (**d**—panel). White arrows in (**a**,**b**) panels indicate the same areas of the specimen.

It is obvious that the formed phases consist of light elements (i.e., they will be difficult to distinguish in BE imaging mode), as well as the complex composition of the layers making their direct analysis using SEM and EDX very difficult. The dissolution of silica-based phases using HF in the manner described above showed that: (i) in the areas where the relief paths and islands were removed, the surface of a carbon film (see EDX analysis: Figure S2a,b, and Table S1, Supplementary Information Section (SIS)), as well as some microscale to nanoscale phases (see Figure 3a,b) were revealed; (ii) in the remaining areas of the substrate surface, the transparent blue/violet-colored layer was completely dissolved and the surface of the Si substrate was exposed; these areas are indicated by “I” in Figure 3c as well as Figure S3a,b (SIF), while the relief features do not change visibly. Further analysis of the relief areas using BS (Figures S4a,b and S5b in SIF) and EDX analysis (see Table S1—SIF) shows that the relief areas are single-phase and most probably consist of carbon. In the BE image (Figure 3b), it can be seen that the small nano-sized particles with a very bright contrast are most likely also composed of carbon phases, on which there are residues of NaOH, with which the patterns were cleaned from the residues of HF: see the results of the EDX analysis—Table S1 and Figure S5b (see SIF).

SEM examination of the II-type films deposited with a much higher content of the carbon-containing precursor in the gas phase reveals that they are phase-homogeneous and contain mainly carbon in crystalline flakes with an area of about 0.5–1 μm^2^ and a thickness in the nanometer range—see Figure 3d.

**Figure 4:**
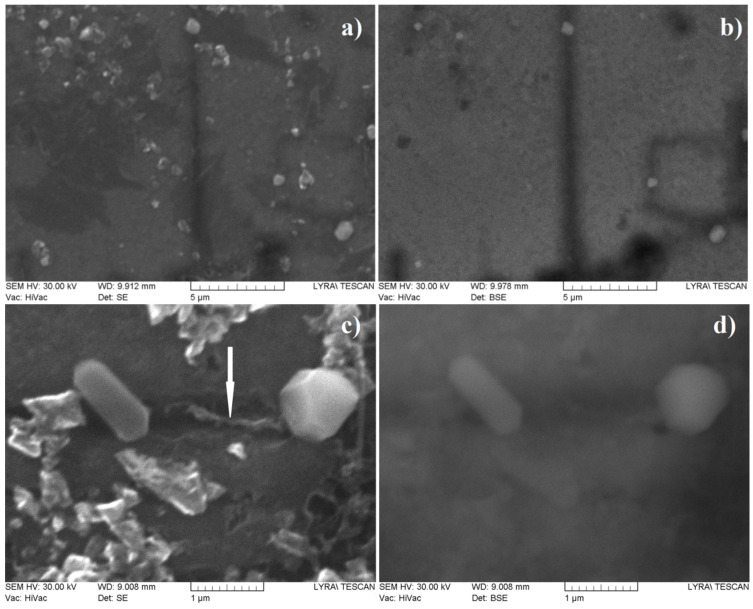
SEM images taken from HF-treated I-type films in SE (**a**,**c**) as well as in BSE (**b**,**d**) image modes. The white arrow in (**c**) panel indicates a graphene flake lying almost perpendicular to the sample surface.

Additional SEM studies at higher magnifications as well as EDX analyses of the areas where the relief formations have been detached from the carbon layer (Figure 4a,b) reveal two distinct features: the first ones are relatively well-shaped prisms with dimensions below 1 µm while the second ones are thin films (with a thickness of less than 100 nm) with tetrahedral hillocks (with sizes up to 100–150 nm) on their upper side. The BE mode images (Figure 4c,d) suggest a different chemical composition of the features that remain after etching in the HF solution. The EDX examinations of the well-shaped clear faceted crystals (Figure 4b,d, as well as Figure S5c and Table S1: see SIF) show that they consist of SiC. By analogy, the thin-layered flakes (some of which are marked with white arrows in Figure 3a,b) are most likely made of SiC. The remaining nano-sized phases have a contrast matching that of the carbon film in the BE image mode (compare Figure 3a and Figure 4a with Figure 3b and Figure 4b) and can therefore be concluded to be of practically pure carbon. In addition, in some areas, very thin and highly folded flakes with sizes up to about 1 μm (one is marked with a white arrow in Figure 4c) are observed, which appear to be multilayer graphene.

### 3.2. X-ray Diffraction (XRD) and Grazing Incidence X-ray Diffraction (GIXRD) Studies

**Figure 5:**
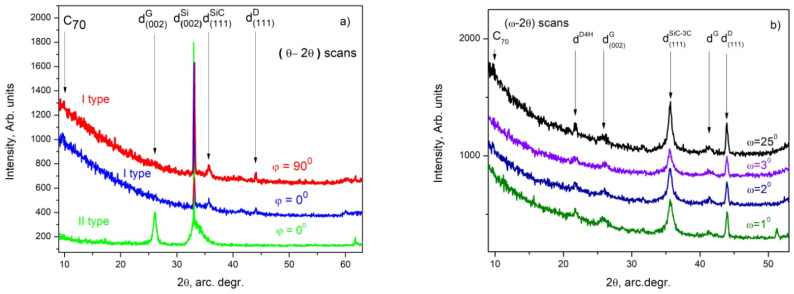
(**a**) Powder XRD patterns (θ–2θ scans) of I-type films (red and blue traces) as well as of II-type (green trace) films taken at φ = 0° (blue and green traces) and at φ = 90° (red trace), respectively; (**b**) GIXRD patterns (ω-2θ scans) taken from I-type films at different values of ω (ω = 1°—olive trace; ω = 2°—royal blue trace; ω = 3°—violet trace; ω = 25°—black trace).

The powder X-ray diffraction (XRD) patterns taken in the θ–2θ scans (Figure 5a) show the presence of cubic diamond peaks denoted by “d^D^”, corresponding to ref. [16] (#ICSD-28857); cubic SiC 3C polytype peaks denoted by “d^SiC^”- [17] (# ICSD-24217); and silicon peaks denoted by “d^Si^” [18] (# ICSD-29287) in specimens of the I type as well as hexagonal graphite peaks denoted by “d^G^”- [19] (#ICSD-31170) in specimens of the II type. Additionally, a weak and broadened peak appears around 2θ = (9.5–10.2)°, indicating the possible presence of C_70_ fullerenes [20] (#ICSD-75506). The 90°- rotation of the specimen of the I type around the [001] axis, i.e., powder X-ray diffraction patterns taken in (θ–2θ) scans at φ = 90°, does not indicate a significant change in the intensity ratio of the main peaks, which shows that texturization of the films does not appear.

All as-deposited films and layers were examined using grazing incidence X-ray diffractometry (GIXRD) examinations in φ scans (0–360)°. The research results do not differ from those presented in our previous work: see ref. [13], which investigated the initial stages of the deposition of carbon phases on a Si substrate using thermally activated CVD and acetone as the precursor. The grazing incidence X-ray diffraction patterns (GIXRD) taken in ω-2θ scans at ω = 1°, 2°, 3° and 25° from specimens of the I type are shown in Figure 5b. The following phases were observed: hexagonal graphite peaks denoted by “d^G^” lie very close to the d_(004)_ of hexagonal graphite [19] (#ICSD-31170); cubic diamond peaks denoted by “d^D^- [16] (#ICSD-28857); cubic SiC 3C polytype peaks denoted by “d^SiC^”- [17] (# ICSD-24217); silicon peaks denoted by “d^Si^” [18] (# ICSD-29287) and diamond polytype peaks denoted by “d^D4H^” [21] (#ICSD-66466). The peak at 2θ = (9.5–10.2)° is distinguishable more or less in scans at ω = 1°, 2°, 3° and 25° and can be ascribed to the XRD pattern of fullerenes C_70_ [20] (#ICSD-75506). No other reflexes were observed in the GIXRD scans taken at ω < 1° (ω = 0.1, 0.25 and 0.5)° and these patterns are not presented here. The d^G^ peak is significantly broadened toward the larger values of d_(004)_ in all GIXRD patterns presented in Figure 3b, as well as in the powder XRD taken from the II-type layers (Figure 3a), which indicates a significant modulation of the interplanar distances between the {001} plane.

The above results clearly show that qualitatively, the I-type films contain several phases: silicon carbide as well as different carbon phases (diamond (including polytypes), sp^2^-hybridized carbon (graphite and derivatives) and most probably C_70_ fullerenes). It can also be concluded that the SiC phase(s) is/are highly textured with respect to the <111> direction for the 3C polytypes of SiC and/or <001> for the hexagonal polytypes 2, 4, 6, etc. The SiC polytypes (only these reflexes are visualized in the diffractograms) and their amounts exceeds that of the other phases in the layers (the reflex at about 35.79° denoted as d^SiC^ in Figure 5a,b is of the strongest intensity).

The II-type layers contain predominantly disoriented multilayered graphene flakes as the reflections of the d_(−110)_, d_(010)_, d_(−111)_, d_(011)_, d_(-112)_ and d_(012)_ planes (see ref. [19] (#ICSD-31170)) are absent. The presence of some amorphized carbon can also be suggested because a significant deviation in the values for d_(002)_ is observed (see the green trace in Figure 3a), and therefore, it can be regarded as highly defective multilayered graphene. The GIXRD studies of the II-type thin layers (Figure S2—see SIF) show that there is definitely no orientational dependence of the deposited layers on the orientation of the substrate, and that the deposited films consist of pyrolytic graphite/multilayered defective graphene (PG) and SiC phases.

### 3.3. X-ray Photoelectron Spectroscopy Studies

**Figure 6:**
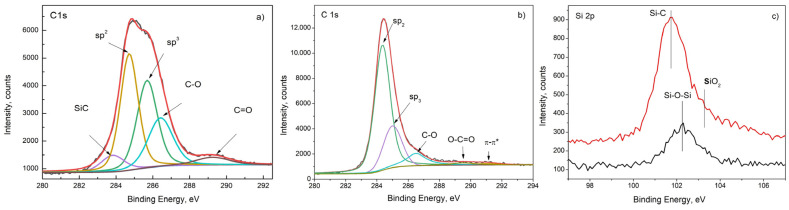
(**a**) Deconvolution of the C1s line of the XP spectrum of I-type film.; (**b**) Deconvolution of the C1s line of the XP spectrum of the II type. (**c**) Si2p peak of I- (red trace) as well as of II-type (black trace) films.

XPS studies were performed in order to improve the chemical composition analysis as well as the phase composition for the I-type as well as in II-type films. The C1s photoelectron line is used for distinguishing the existing carbon bonds and thus it has been deconvoluted (the results are presented in Figure 6a,b). The C1s photoelectron spectra measured in the I–type films show the formation of SiC (the peak at 283.2 eV is the evidence for the formation of SiC [22,23]) in addition to the sp^2^, sp^3^, C-O and C=O bonds that are present as well. The sp^3^/sp^2^-hybridized carbon ratio is 1.17. The analysis results of the Si2p spectra confirmed a Si–C binding energy of 101.8 eV, as well as the presence of SiO_2_ (see Figure 6c red trace). The SiO_2_ content is low while the Si–C bonding is high, which verifies that the high carbon content competed with the Si–O bonding as carbon atoms entered into a more stable oxygen site to become SiC, as was established earlier [24].

The C1s photoelectron spectra of the II-type sample are typical for defective graphene, graphite and pyrolytical graphite (PG) (see [13,25]). The maximum of the peak appears at 284.4 eV and it is typical for a sp^2^–carbon bond. At the asymmetrical tail at the higher binding energy side of the peak maximum, additional bonds exist associated with sp^3^, C–O, O–C=O and π–π*. The presence of a π–π* bond is clear evidence for predominant sp^2^-type bonds in the carbon film. The calculated sp^3^/sp^2^ ratio in the sample is 0.34. The peak of Si2p for the II-type film (Figure 6c- black trace) appears at 102.3 eV and we associate it with the formation of SiO_x._

### 3.4. Raman Spectroscopy Studies

Raman spectroscopy is a powerful method for the qualitative analysis of carbonaceous phases as shown in many references: the Raman spectrum of graphene can serve as a fingerprint [26]. Graphite has also unique Raman spectrum [27,28]; the sp^2^/sp^3^ ratio of the mixed amorphized carbon phases can be determined using Raman spectroscopy [29]. Diamond can also be identified using Raman spectroscopy [30,31,32,33], etc.

The etched surface of the I-type films is inhomogeneous and uneven; thus, the Raman spectrum was measured in different points denoted by 1, 2 and 3 in Figure 2b. The observed Raman peaks and bands can be ascribed to D, G, D’ and 2D, which are the typical Raman bands of graphene [26]; D^Σ^- Raman peaks of: D^3^ diamond [23,24,25,26] and D^1^ and D^2^ diamond polytypes [34,35]; the SiC1, SiC2, SiC3 and SiC4 SiC polytypes [36]; CH and 2CH hexagonal *n*-C_36_H_74_ polyethylene chains [37] as well as (C_1_, C_2_ and C_3_) carbon-based chains of polylactic acid and polyethylenes [38].

**Figure 7:**
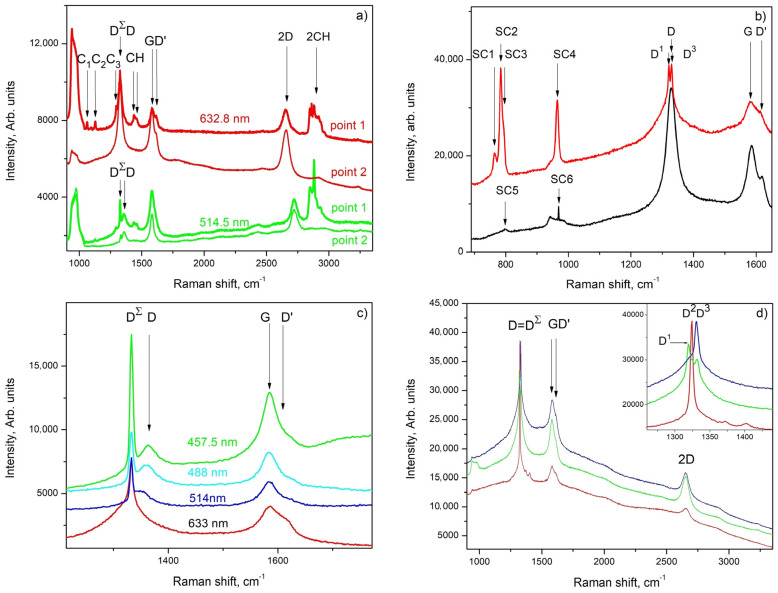
(**a**) Raman spectra of a I-type film after the etching procedure measured in points 1 and 2 (Figure 2b) using 633 nm (red traces) and 515 nm (green traces); (**b**) Raman spectra (633 nm excitation wavelength) of PG intermediate film with some nano-sized SiC crystals; (**c**) one-phonon Raman spectra of PG films as well as of diamond crystals measured using 633 (red trace), 514 (blue trace), 488 (cyan trace) and 458 (green trace) nm excitation laser wavelengths, respectively; (**d**) Raman spectra (633 nm excitation) of diamond polytypes.

**Figure 8:**
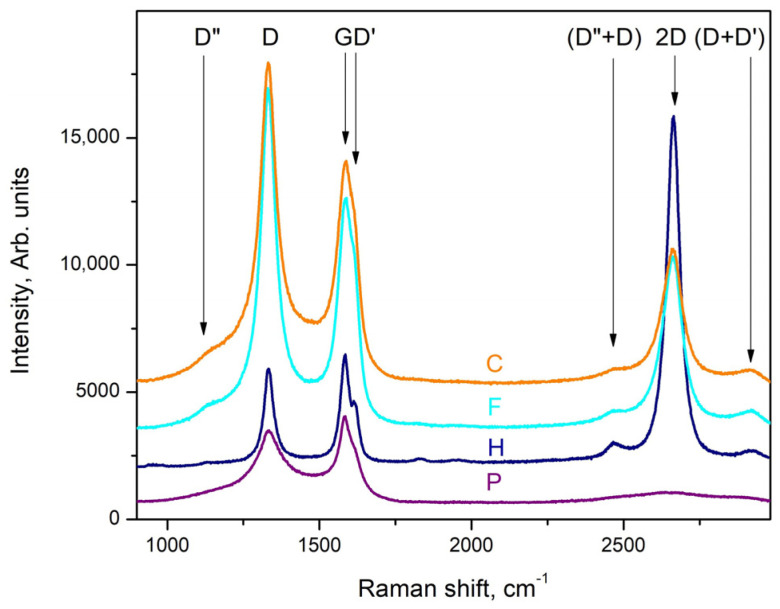
Raman spectra measured at different points in II-type carbon films (see Figure 2d): point denoted by P purple trace, point denoted by H navy blue trace, point denoted by F cyan trace and C (orange trace) a point in the middle part of the 2-inch {001} Si substrate.

According to these results and recalling the results presented in [12,13], it can be assumed that the surface of the sample is covered with a very thin graphene-like layer containing mainly of sp^2^-hybridized carbon and small amounts of sp^3^-hybridized carbon and carbon chains of polyethylenes (see the Raman spectrum measured in point 1 (Figure 7a)). It also seems that in its thicker parts, the layer becomes similar to defect-rich multilayered graphene (see the Raman spectrum taken from point 2 (Figure 7a and Figure 8)). Also, due to the double-resonant origin of the D-band in graphene/graphene-like materials and graphite (see ref. [29]), in our case, one can very easily distinguish the contribution of the D-band of graphite/graphene and the D-peak of diamond by varying the wavelength of the exciting laser light (Figure 7c) and undoubtedly show that both are present in the investigated samples.

Various features that can be related to the Raman spectrum of SiC are measured near (or directly on) dark lines similar to those observed in Figure 2b, Figure 3a,b and Figure 4a–d whereas in Figure 7b, only those features that can be assigned to different SiC polytypes are shown. Several peaks distinguishable in Figure 7b can be unconditionally attributed to SiC and they are denoted by SiC1 (at about 766 cm^−1^), SiC2 (at about 784 cm^−1^), SiC3 (at about 797 cm^−1^) and SiC4, with a maximum at 965 cm^−1^, but visibly broadened toward the lower wavenumbers of the spectrum, represented by the red trace in Figure 7b, most likely belong to the 2H and 6H polytypes of SiC (see [36]), while SiC5 (at about 797 cm^−1^) and SiC6 (at about 970 cm^−1^) from the spectrum represented by the black trace in Figure 7b should be ascribed to the 3C polytype of SiC [36].

Another important result is the measurement of Raman spectra at different points in the area marked with 3 in Figure 2b; they show an apparent change in the frequency of the strongest diamond peaks. Complex diamond D-peaks are denoted together by “D^Σ^” in Figure 7a–d, while in the inset of Figure 7d, individual peaks are denoted by D^1^, D^2^ and D^3^. The individual diamond peaks appear at 1321 cm^−1^, 1326 cm^−1^ and 1332 cm^−1^, respectively. The observation of diamond peaks at a wavenumber lower than 1332 cm^−1^ can be regarded as a clear indication of the presence of diamond polytypes in the studied sample [34,35]. The latter thus confirmed the result of the GIXRD (Figure 3b), where a peak characteristic of diamond polytypes (see [21] #ICSD-66466) is distinguished. It should be also noted that peaks D^1^ and D^3^ also appear in the spectrum presented in Figure 7b.

The Raman spectra measured in the II-type layers are typical of defect-rich multilayered graphene: the D–band is dominating, the G and D’ bands have similar intensities and an almost symmetrical 2D band is clearly defined (its intensity is 50–60% of that of the G-band). The observed defects of the hillock-centered crater type (Figure 2c,d) allow access in close proximity to the substrate. The Raman spectrum measured at point P, i.e., exactly in the central part of the hillock (purple trace in Figure 8), is typical for amorphized graphite (sp^2^-hybridized carbon); the one measured at point H (navy blue trace in Figure 8) is typical for single-layer graphene with defects (the intensity of the 2D band is 5–6 times larger than that of the G–band, but the 2D band is slightly shifted from 2640 cm^−1^ to 2658–2660 cm^−1^ and all “defect”–related bands [25,39], i.e., D, D’ and D”, are clearly pronounced: see, for example, [12,13]) and the spectrum measured at point F (cyan trace in Figure 8) is very similar to that of the middle part of layer (orange trace in Figure 8) and is typical for PG (pyrolytical carbon/multilayered graphene).

### 3.5. Theory/Calculations

The results presented above, i.e., the parallel deposition of carbon phases with predominant sp^2^ hybridization, as well as those with predominant sp^3^ hybridization under very similar conditions, clearly outline two problems: how and why this happens, and whether sp^2^ (sp^3^)-hybridized carbon grow on a carbon surface with sp^3^ (sp^2^) hybridization. We believe that the solution of such tasks should be carried out with the help of quantum-chemical calculations. In this regard, it is clear to us that ab initio simulations of stable nucleation of sp^2^ (resp. sp^3^)-hybridized carbon nuclei/us demand high computation resources. On the other hand, there is no doubt for us that the stable deposition of sp^2^-hybridized carbon on a Si substrate from the products of the thermal dissociation of acetone at 1150 °C is feasible, including in our work the successful deposition of graphene/graphene-like phases using this methodology has been reported; see [5,6]. Accordingly, we decided to limit ourselves to simulations of the stable growth of a sp^2^-hybridized (resp. sp^3^-hybridized) phase due to the thermally stimulated dissociation of acetone on the seed of a sp^2^-hybridized (resp. sp^3^-hybridized) carbon phase. Accordingly, we decided that the deposition reactions of these phases on the corresponding resistant seeds should be simulated under the conditions of the experiments presented above (thermally stimulated decomposition of acetone at a temperature of ~1150 °C and ambient pressure).

At the experimental temperature, acetone decomposes into methane and ketene, while ketene decomposes into methane and ethane [40,41]. Among the major decomposition products of ethene are methane, acetylene and carbon [42,43]. The most probably source (and by far) of carbon yield is the acetylene [43]. Its dehydrogenation results in the highly reactive *C≡C* (denoted hereafter by “C_2_^”^) radical.

The starting structure for the growth of the sp^2^ carbon phase is to obtain a single-layered graphene sheet with defects. The elementary cell of graphene is repeated six times in the direction of edges a and b. The atom vacancies are introduced into the perfect graphene sheet and it finally contains 61 atoms.

To demonstrate the growth of a carbon sp^3^ phase, the goal is a fragment formation containing carbon atoms in sp^3^ hybridization on top of the {111} plane of diamond. The supercell is a 2D structure with an explicit cell of 48 carbon atoms arranged in a diamond configuration, with two {111} surfaces (2 × 1/3 (111) of a diamond cell) open in the third dimension. The opposite (111) surface (i.e., (-1-1-1)) is passivated using hydrogen atoms to avoid extreme reactivity and to stabilize the structure. All the atoms of the initial sp^3^ structure have fixed positions.

The temperature of the simulations is 1438.15 K. The thermostat is a Canonical Sampling through Velocity Rescaling (CSVR) thermostat. The timestep is 1 fs. All MTD are in the NVT ensemble. Then the height if there are Gaussian penalty potentials (hills) is 5.251 kJ/mol. The scale factor (Gaussian width) for each collective variable (CV) is 0.2. Hills are spawned every 50 fs. Walls, when used, are of the quadratic type and with a potential constant of 83.68 kJ/mol. The temperature tolerance is always 100 K. The shortest distances between reacting atoms in the initial steps are no less than 300 pm.

The set colvars (CV) are C—C distances. Example transition state (TS) geometries are chosen from the trajectories as the most representative configurations for the simulated reactions.

At the experimental temperature, acetone decomposes into methane and ketene, and thereafter, ketene decomposes into methane and ethene [40,41]. Among the major decomposition products of ethene are methane, acetylene and carbon [42,43]. The most probable carbon yield source is acetylene [43]. Its dehydrogenation results in the highly reactive C≡C (denoted hereon as C_2_) radical.

#### 3.5.1. Deposition of the sp^2^-Hybridized Carbon Phase

**Figure 9:**
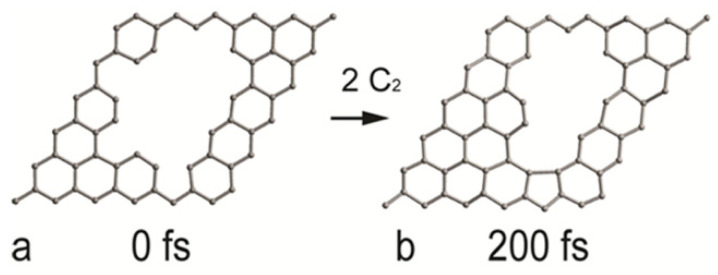
Defective graphene sheet (**a**) and the product of sp^2^ carbon deposition (**b**) with C_2_ radicals.

The starting structure for the growth of the sp^2^ carbon phase is a single-layered graphene sheet with defects: see Figure 9a. During the MTD simulation, two C_2_ radicals are added to the system in order to accomplish new six-membered rings, as in Figure 9b. The whole process takes 200 fs.

#### 3.5.2. Deposition of the sp^3^-Hybridized Carbon Phase

Multiple sequential MTD simulations are used to demonstrate the deposition of sp^3^ carbon phase on a {111} plane of diamond. The initial geometry of each synthetic step is either a frame from a previous step or the optimized product of a previous step. One source of carbon is methane. The complete dehydrogenation of a methyl group attached to the diamond structure is included as a synthetic step. In total, the final product of sp^3^ carbon deposition is with simulated reactions between the attached C atoms, additional C_2_ radicals and the diamond {111} surface.

**Figure 10:**
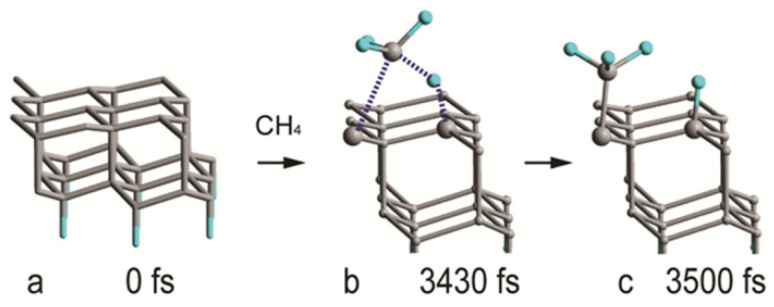
Geometries of the critical structures of the reaction between diamond (111) surface and methane: (**a**) initial geometry, (**b**) transition state, (**c**) product.

**Figure 11:**
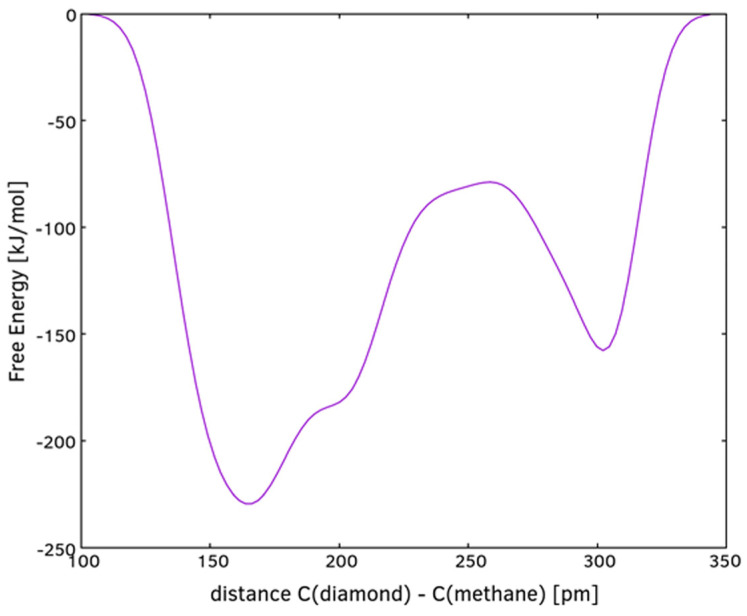
Free energy profile of the reaction between methane and {111} diamond surface.

Multiple sequential MTD simulations are used to demonstrate the deposition of the sp^3^ carbon phase on a {111} plane of diamond. The first MTD run of the sequence is a reaction between the diamond {111} plane and methane. The initial frame is shown in Figure 10a. A transition state (TS) is reached at 3430 fs. One of the methane hydrogen atoms binds to a surface C atom, simultaneously with the formation of a C—C bond between the methane C and the surface (Figure 10b). The forward free energy barrier of the reaction is 80 kJ/mol (Figure 11). The reverse barrier was not completely studied and is larger than what Figure 11 depicts. The final product (methylated diamond surface) is shown in Figure 10c.

**Figure 12:**
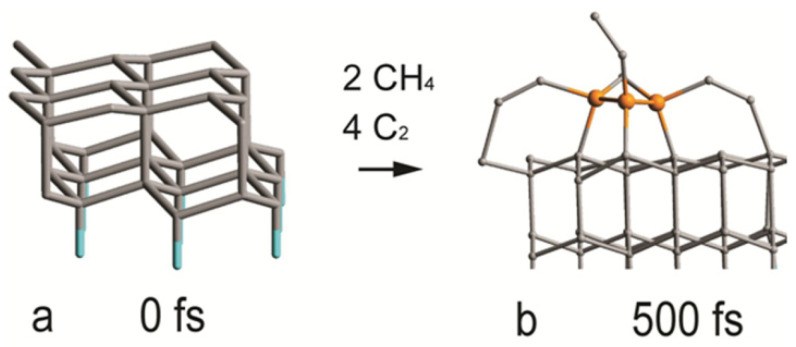
Geometries of the initial (**a**) and final (**b**) structures of the synthesis of the growth of sp^3^ carbon phase.

The geometry of the initial 2D diamond structure cut by the {111} plane is presented in Figure 12a. The MTD synthetic steps include the addition of a methyl group from methane, dehydrogenation and multiple steps of C_2_ addition. The C_2_ additions are barrierless. The geometry of the final formation is presented in Figure 12b. Clearly visible are three carbon atoms in the sp^3^ state, demonstrating the growth of a sp^3^ phase on top of the surface of the initial sp^3^ structure.

The possibility of the synthesis of a 2D/3D carbon phase with sp^3^ hybridization on a carbon phase with sp^2^ hybridization was not established. Cell optimization is applied to the final structures of the three syntheses, in order to find the energetic effects. All three possible routes are exothermic (Table 1). The formation of a sp^2^ phase is expected to be energetically preferred, as it requires only one carbon donor reagent (and it is the highly reactive one), as well as a smaller number of geometrically simpler steps for it to occur.

## 4. Discussion

The presented experimental results for the deposition of carbon phases at temperatures around 1150 °C allow us to draw some conclusions about the synthesis path, as well as about the most probable sequence of the ongoing processes:

- We found that the change in the concentration of the hydrocarbon precursor in the gaseous phase affects very strongly the phase composition of the deposited carbon phases: the relatively high concentration of the precursor (120 cm^3^/min at 18 °C (Ar + C_3_H_6_O) flow) provides a high supersaturation of the gas phase and a relatively high rate of deposition of the carbon layer, in the order of 300–450 nm/h, and the deposited films are of multilayer defective graphene flakes with an area of up to 1 μm^2^, which may be referred to as pyrolytic graphite, while the low gas phase precursor concentration (about (30 cm^3^/min (Ar + C_3_H_6_O) + 150 cm^3^/min Ar) gas flow) ensures a low deposition rate (below 100 nm/h) and the formation of various nano-sized carbon phases: flakes of defective graphene, nanodiamonds and various polytypes of the diamond;
- In the thin films deposited at a low concentration of acetone in the Ar main gas flow, a phase of SiO_2_ (or SiO_2_-based glass of variable composition, i.e., Si_(1−x)_C_x_O_(2−y)_) was found to be deposited in parallel to a Si substrate occupying on average approximately 5% of the substrate surface, and a shiny and transparent relief layer, whose Raman spectrum matches that of amorphous carbon (a mixture of sp^2^-hybridized and sp^3^-hybridized carbon) on the remaining ~95% of the substrate’s surface The two layers were relatively thick (about 100 nm) in each of the 60 and 90 min deposition time experiments.. On the surface of the amorphous carbon films, there are formed micro-sized layered crystals, very similar to those described in our previous works (see [14]), some of which in certain positions have a Raman spectrum matching that of diamond. Varying the deposition time (within 45–90 min) of the synthesis processes for this type of layer generally does not lead to a change in the phase composition and morphology of the obtained layers and affects only the thickness of the layers, as the deposition rate of these layers is about 50–65 nm/h. It can be assumed that the natural oxide covering the Si substrate is mostly released with the help of carbon atoms, reaction products of the thermally stimulated decomposition of acetone. The rest of the oxygen atoms can be assumed to migrate in the form of -CO/-SiO on the Si surface until the formation of a small area of SiO_(2−x)_/Si_(1−x)_C_x_O_(2−y)_ regions;
- In the thin films deposited at a low concentration of acetone in the Ar main gas flow, a thin intermediate carbon film of predominantly sp^2^-hybridized carbon (averagely 80%) on the Si substrate was observed using SEM (gray area in Figure 3a,b and Figure 4a–e) and Raman spectroscopy (Figure 7a–e). Judging from the Raman spectra, these films mainly contain multilayer defective graphene flakes as well as saturated aliphatic (sp^3^- hybridized carbon) chains (n-CH_2_-) in significant amounts (15 to 20% according to XPS measurements) and most likely small amounts of C_70_ fullerenes. Such a scenario (the formation of a mixed film of sp^2^- and sp^3^-hybridized carbon on a Si substrate in the first stages of the process) is also supported by the results of the molecular dynamics simulations. As was clarified in the Theory/calculations section, as far as the deposition reaction of sp^2^-hybridized carbon from the C_2_ radicals has the most significant energetic effect (ΔE = −1882 kJ/mol), then the amount of deposited sp^2^-hybridized carbon will be much greater than that of the sp^3^-hybridized carbon 3D phase deposited from (1 × CH_4_) molecules (ΔE = −352 kJ/mol) or from (2 × CH_4_) molecules + (4 × C_2_) radicals (ΔE = −1453 kJ/mol). Furthermore, as the synthesis processes progressed, single-phase nano-sized diamonds and “hexagonal” diamond crystals (diamond polytypes) grew on some of the regions of predominantly sp^3^-hybridized carbon from the intermediate carbon film;
- The XRD and GIXRD studies showed that regardless of the deposition mode, the deposited layers always contained SiC (see GIXRD and XRD diffraction patterns—Figure 5a,b and Figure S1). Since the successful synthesis of this phase requires the access of Si atoms to the reaction surface, it can be reasonably assumed that this process always takes place at the interface between the Si substrate surface and the deposited carbon film. On the other hand, there are many experimental results (see, e.g., refs. [44,45,46,47]) showing that at these temperatures, 1000–1200 °C, the deposition of carbon on a Si substrate always leads to the partial or complete carbonization of the Si surface. Moreover, recently in our work (see ref. [48]), we have shown that at temperatures around 1150 °C, the decomposition of methane on a Si substrate always produces SiC, with a large fraction of native oxide migrating along the surface of the substrate. According to the considerations so far, it can be concluded that in the first stages of the deposition, a SiC film (at the Si substrate/carbon layer interface) is formed. It can also be assumed that at a low concentration of the hydrocarbon precursor, the rate of deposition of the carbon layer (at least in some areas) does not exceed the rate of synthesis of SiC, i.e., there are conditions for the deposition of only the SiC film. In the next stage, after the formation of SiC with a thickness of more than several tens of nanometers, as well as when using high concentrations of acetone in the gas phase, the deposition of the carbon layer, which was discussed in the previous paragraph, begins on the SiC film. This occurs because the formation of a SiC film depends on the possibility of the diffusion of Si atoms from the substrate to the interface, i.e., of the thickness of the SiC layer and the temperature, and sooner or later, the process will end, due to the weak flow of Si atoms through the SiC. Regarding the further deposition process, SiO_2_/SiO_(2−x)_ regions grow due to changes in the composition of the oxide film, “dissolving” small amounts of Si from the substrate and probably joining some -CO radicals from the dissociated acetone. We should emphasize that the simulation results of these processes (see ref. [48]) confirm a similar behavior in the oxide precipitates; simulations showed that under these conditions, it is impossible to deposit 3D sp^3^-hybridized carbon (diamond and polytypes based on it) on 2D sp^2^-hybridized carbon (graphene and graphene-like phases;
- And finally, the most unclear seems to be the synthesis of nano-sized crystals (a volume of up to several hundreds of nanometers) (see Figure 3a,b, Figure 4a–d and Figure S3c) because they grow on the carbon intermediate layer discussed above. However, in the mentioned SEM images, it can be seen that the SiC crystals are always arranged on strip-shaped voids oriented parallel to <011> (see Figure 3b and Figure 4b), i.e., it can be assumed that they were occupied by a phase that was dissolved by HF, most likely based on SiO_2_, i.e., through it, it was possible to feed the growing phase with the Si from the substrate.

We believe that acetone for several reasons (relatively safe organic chemical with a sufficiently high vapor pressure at temperatures around room temperature, close to the linear dependence of saturated vapor pressure on temperature in the interval 12–24 °C, etc.) is a convenient but highly underestimated precursor for the synthesis of carbon phases using CVD processes based on its thermally stimulated decomposition. Considering the possibilities of using in practice CVD processes from a gas phase containing acetone vapor:

(A) It can be assumed that deposition processes using gas phase hydrocarbon precursor concentrations lower than those used in the experiments considered here will lead to the synthesis exclusively of sp^2^-hybridized carbon phases, including graphene/graphene-like phases, which is confirmed by our previous research [12,13]. This conclusion is also confirmed by the measurement of the Raman spectra of defective graphene on the areas with SiO_2_ at the bottom of hillock-centered craters in the II-type layers. In these areas, there is an extremely low feeding flow of carbon from the gaseous phase: see Figure 2c,e and Figure 8;
(B) On the other hand, the synthesis of only a sp^3^-hybridized carbon phase seems a bit more complicated, but we can assume that it is quite possible to occur using sp^3^-hybridized carbon phase nuclei, e.g., nano/microcrystalline diamond powder, and a similar conclusion is also supported by our molecular dynamics simulations: see the Theory/calculations section and Table 1;
(C) The results presented here show that deposition processes of carbon phases on Si substrates via CVD processes from the acetone vapor in the carrier gas argon at temperatures around 1150 °C can be used to deposit thin layers of SiC.;
(D) In addition, the possibility of parallel synthesis of carbon nanophases became very relevant after it was recently shown that amorphous carbon can be modified into nanodiamond nanocomposites embedded into multilayer graphene with unique properties: see ref. [47].

## 5. Conclusions

The deposition of carbon films using CVD processes based on the thermal decomposition of acetone performed at high temperatures of about 1150 °C in a main flow of Ar at different concentrations of the carbon-containing precursor and a relatively long deposition time (in the range of 45 to 90 min) were studied. The I-type films were deposited using a low concentration of the carbon precursor in the main Ar stream, while a fourfold higher concentration was used to deposit the II-type films. The films obtained in these processes are different: in the first case, a layer consisting mainly of nanocrystalline/amorphous carbon and partially (up to 5% of the total area of the layers) of silicon oxide is formed; and in the second case, a layer of microcrystalline pyrolytic graphite is formed. In order to be able to reveal the phases in depth of the I-type layers, they were treated using a 15% HF solution. The thus obtained samples of type I as well as those of type II were examined using SEM (by a LYRA TESCAN, TESCAN GROUP a.s., Brno, Czech Republic) or a JEOL JSM-6390 microscope, JEOL Ltd., Tokyo, Japan), XRD and GIXRD (by Panalytical Emperian apparatus, Malvern Panalytical, Malvern, United Kingdom), X-ray photoelectron spectroscopy (by a VG ESCALAB (Thermo Fisher Scientific, Waltham, MA, USA) and Raman spectroscopy (by a micro-Raman HORIBA Jobin Yvon aparatus (HORIBA Jobin Yvon, Longjumeau, France). Deposits of SiC nanocrystals, diamond phases (including diamond polytypes) and defective graphene/thin pyrolytic carbon films were found in the studied samples.

Additionally, several conclusions regarding the pathways for the deposition of the carbon phases on the Si substrates can also be derived. The layer deposition process is always associated initially with the formation of a SiC film via the direct carbonization of the surface of the Si substrate. Further on, either 2D sp^2^-hybridized carbon in the form of graphene-like (few-layered to multilayered defective graphene) phases, or 3D sp^3^-hybridized carbon (diamond and/or so-called hexagonal diamond) is deposited locally on the SiC thin films. In the subsequent stages of such processes (necessary conditions include a relatively long (>45 min.) deposition process and available native oxide on the Si surface before the start of the CVD process), the surface of the samples (on which there is silicon oxide remaining) is covered with SiO_2_-enriched films and C–O–C complexes, whereas the rest of the sample surface is covered with a film structurally similar to aC. Such films likely result from the migration of the SiO_2_ and Si–O–C complexes in the SiC/carbon films to and from the isolated areas occupied by SiO_2_.

## Figures and Tables

**Table 1:** Estimated energetic effects (in kJ/mol) of the three deposition paths: deposition of sp^2^-hybridized carbon via *C≡C* (C_2_) radical/s; deposition of sp^3^-hybridized carbon phase via CH_4_ and deposition of sp^3^-hybridized carbon phase via 2 × CH_4_ + 4 × C_2_ radicals.

Simulation	Energetic Effect [kJ/mol]
Deposition of sp^2^ hybridized carbon phase, via *C≡C* (C_2_) radical/s	−1882
Deposition of sp^3^-hybridized carbon phase, via CH_4_	−352
Deposition of sp^3^-hybridized carbon phase, via 2CH_4_ + 4C_2_ radicals	−1453

## Data Availability

Data is contained within the article.

## Supplementary Materials

The following supporting information can be downloaded at https://www.mdpi.com/article/10.3390/ma16227190/s1. Figure S1. Raman spectra (RS) taken from different positions of the relief area of the as-grown films of I-type: RS taken from graphite films similar to defected multi-layered graphene area of the film (navy trace) to RS from a more amorphized defected multi-layered graphene area of the film (royal blue trace). Figure S2. GIXRD patterns (ω-2θ scans) taken from II-type films at different values of ω (ω = 10 and φ = 00-magenta trace; ω = 10 and φ = 900-orange trace; ω = 30 and φ = 000-dark grey trace). Figure S3. (a,b) Secondary electron (SE) images taken from the relief area after HF treatment. Figure S4. (a) SE image taken from the round island with a relief surface similar to those shown in Figure S2a. A4 (b) Backscattered electron (BSE) image taken from the same area as those shown in Figure S4a. Figure S5. (a–c). SE images of the points examined by EDX analysis (the “+” positions of the images). See Table S1 for summarized results of EDX analysis. Table S1. EDX analysis taken from points remarked by “+” in Figure S5a–c. Ref. [49] is cited in the supplementary file.
