# Supplementary material for: Study of the Chemical Vapor Deposition of Nano-Sized Carbon Phases on {001} Silicon
Source: Materials (Basel). 2023 Nov 16;16(22):7190. doi: 10.3390/ma16227190 (PMC10672876; doi:10.3390/ma16227190)
Supplement: Supplementary file 1 [file materials-16-07190-s001.zip › materials-2705275-supplementary.pdf]

Supplementary Information File

1) **Raman spectra taken from the relief area of the as- grown films from I- type specimens**

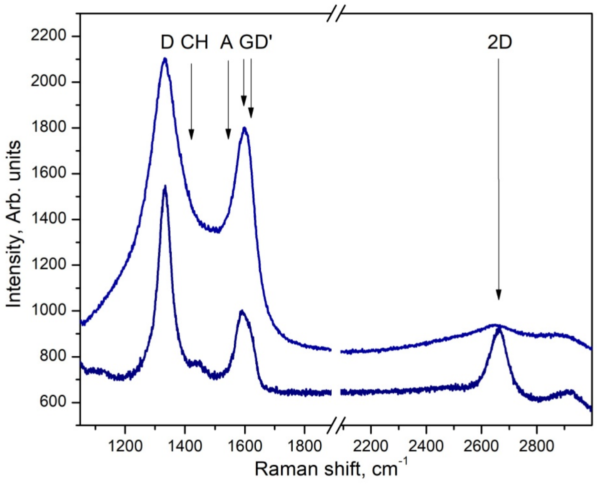

Figure S1. Raman spectra (RS) taken from different positions of the relief area of the as- grown films of I- type: RS taken from graphite films similar to defected multi-layered graphene area of the film (navy trace) to RS from a more amorphized defected multi-layered graphene area of the film (royal blue trace).

The relief transparent areas (the areas denoted by "II" in Figure 3a,b) have a clear Raman spectrum that varies in different positions. However, the observed features are common for all Raman spectra measures and can be defined as the well-known graphite/graphene/amorphous carbon features: D, G and D′ bands (see Refs. [1–4]). The CH- denoted feature (at about 1460 cm^{-1}) is usually related to hexagonal n-$C_{36}H_{74}$ polyethylene chains [5]. An additional band (denoted by A and arising at about 1540 cm^{-1}) which is attributed to oxygen- containing carbon radicals bonded to the edges/ vacancies of the graphene/ graphite sheets is clearly distinguished- see ref. [6]. Since the intensity of the CH and A bands is enhanced, and all the other bands are broadened to the extent that the D and G bands merge at their bases, it can be concluded that the amorphous content in these regions increases in the order of the navy blue colored to the royal blue colored trace- see Figure S1.

2) Grazing incidence X-ray diffraction patterns (GIXRD) taken in (ω- 2θ) scans at ω= 1⁰ and 3⁰ from II-type specimens

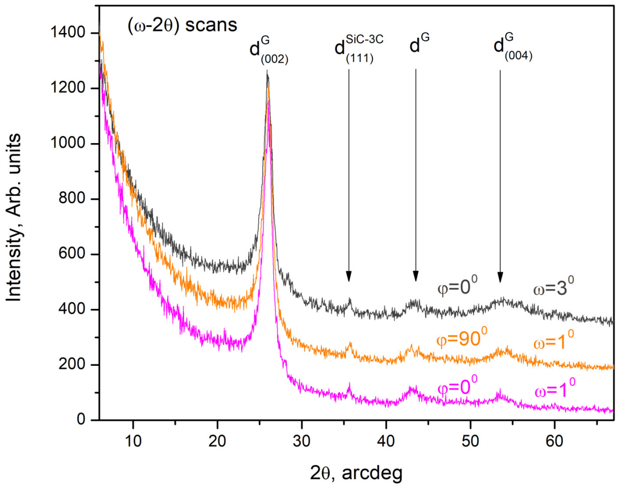

Figure S2. GIXRD patterns (ω- 2θ scans) taken from II- type films at different values of ω (ω=1⁰ and φ=0⁰- magenta trace; ω=1⁰ and φ=90⁰-orange trace; ω=3⁰ and φ=00⁰- dark grey trace).

3) SEM and energy- disperisive X-ray analysis study of I-type thin films

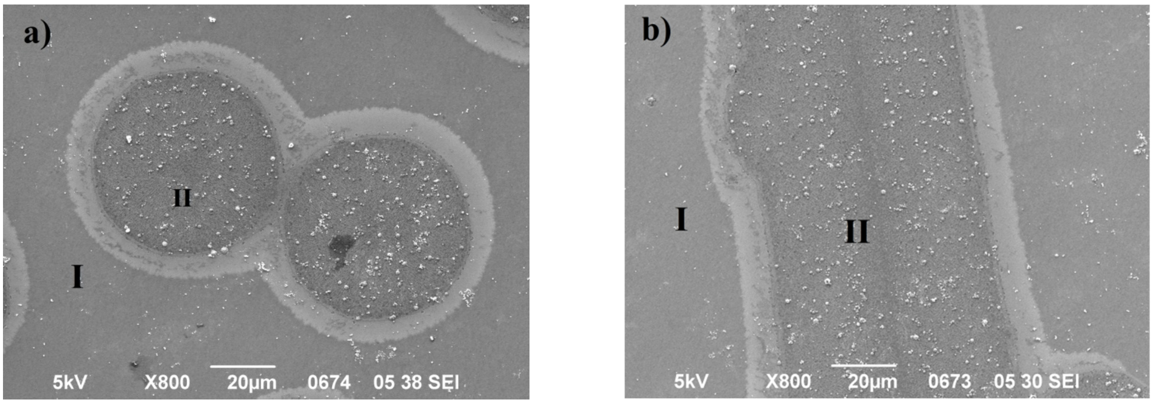

Figure S3. (**a**,**b**) Secondary electron (SE) images taken from the relief area after HF treatment.

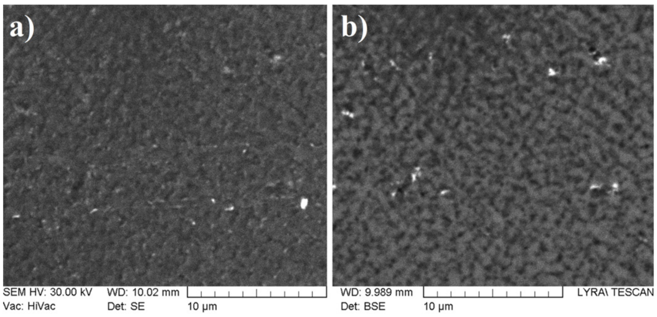

Figure S4. (**a**) SE image taken from the round island with a relief surface similar to those shown in Figure S3a. (**b**) Backscattered electron (BSE) image taken from the same area as those shown in Figure S4a.

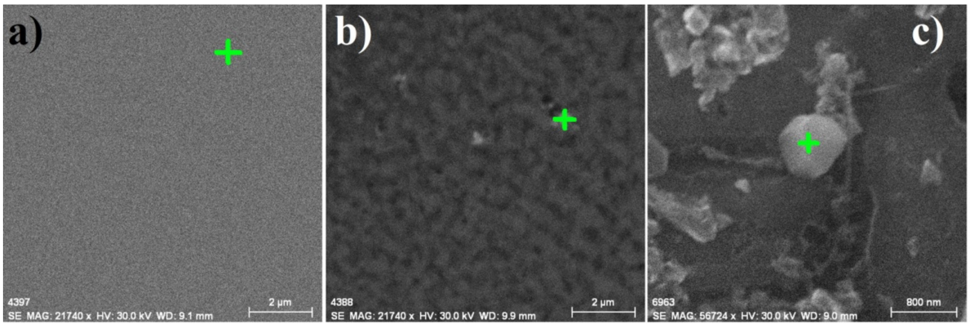

Figure S5. (**a–c**). SE images of the points examined by EDX analysis (the "+" positions of the images). See Table S1 for summarized results of EDX analysis.

Table S1. EDX analysis taken from points remarked by "+" in Figure S5a–c.

**Point, denoted by "+" in Figure S4**		**Point, denoted by "+" in Figure S4**		**Point, denoted by "+" in Figure S4**	
Element	Atomic %	Element	Atomic %	Element	Atomic %
C	20.96	C	44.07	C	50.12
Si	70.52	Si	40.02	Si	49.88
O	8.53	O	15.91	-	-

References

1. A.C. Ferrari and D.M. Basko, Nat. Nanotechnol., Raman spectroscopy as a versatile tool for studying the properties of graphene, 8 (2013) 235. https://doi.org/10.1038/nnano.2013.46
2. F. Tuinstra and J. L. Koenig, Raman spectrum of graphite, The Journal of Chemical Physics, 53 (1970) 1126. https://doi/10.1063/1.1674108
3. C. Thomsen and S. Reich, Double Resonant Raman Scattering in Graphite, Phys. Rev. Lett. 85 (2000) 5214. https://doi.org/10.1103/PhysRevLett.85.5214
4. A.C. Ferrari and J. Robertson, Raman spectroscopy of amorphous, nanostructured, diamond–like carbon, and nanodiamond, Phil. Trans. R. Soc. A 362 (2004) 2477. https://doi.org/10.1098/rsta.2004.1452
5. R. G. Snyder, S. L. Hsut and S. Krimm, Vibrational spectra in the C-H stretching region and the structure of the polymethylene chain, Spectrochim. Acta 34A (1978) 395 https://doi.org/10.1016/0584-8539(78)80167-6
6. S. Claramunt, A. Varea, D. Lopez-Diaz, M.M. Velázquez, A. Cornet and A. Cirera, The Importance of Interbands on the Interpretation of the Raman Spectrum of Graphene Oxide, J. Phys. Chem. C 119 (2015) 10123 https://doi.org/10.1021/acs.jpcc.5b01590
